# Supplementary material for: N-Acetyl Serotonin Alleviates Oxidative Damage by Activating Nuclear Factor Erythroid 2-Related Factor 2 Signaling in Porcine Enterocytes
Source: Antioxidants (Basel). 2020 Apr 7;9(4):303. doi: 10.3390/antiox9040303 (PMC7222184; doi:10.3390/antiox9040303)
Supplement: Supplementary file 1 [file antioxidants-09-00303-s001.zip › antioxidants-737963-supplementary.docx]

**Supplementary materials**

**Supplementary Fig. S1.** Morphological alteration of cells. IPEC-1 cells pre-treated with or without NAS (0- 250 μM, 12 h) were subjected to 4-HNE (20 μM) or DMSO for 2 h. Morphological alteration of cells were observed by using a phase contrast microscopy, magnification ×100.


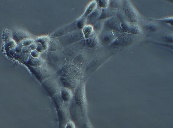

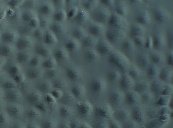

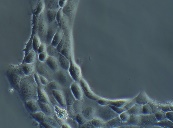

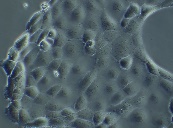

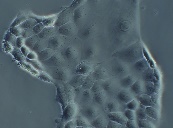


**4-HNE+**

**NAS (250 μM)**

**4-HNE+**

**NAS (100 μM)**

**4-HNE+**

**NAS (50 μM)**

**4-HNE**

**Ctrl**

**Supplementary materials**

**Supplementary Fig. S2.** Protein band density analysis of N-Nrf2, T-Nrf2, Bax, Bcl-2, GSS, HO-1, NQO1, and GCLC. Cell were treated as in Fig.5. Statistical analysis for protein density was determined. Values are means ± SEMs, n = 3. Means without a common letter differ, *P* < 0.05.
